# PPNet: a deep learning framework for rice seedling localization and distribution from UAV orthomosaics

**DOI:** 10.3389/fpls.2026.1932877

**Published:** 2026-10-13

**Authors:** Yongchao Shan, Anqi Zhang, Xiaofei An, Jianhui Nie, Zhicheng Zhang, Chunjiang Zhao, Jingping Chen, Weiqiang Fu

**Affiliations:** 1 College of Information and Electrical Engineering, China Agricultural University, Beijing, China; 2 Research Center of Intelligent Equipment, Beijing Academy of Agriculture and Forestry Sciences, Beijing, China; 3 State Key Laboratory of Intelligent Agricultural Power Equipment, Beijing, China; 4 Research Center of Information Technology, Beijing Academy of Agriculture and Forestry Sciences, Beijing, China

**Keywords:** deep learning, rice seedling counting, rice seedling localization, spatial distribution, UAV orthomosaics

## Abstract

Field-scale information on rice seedling distribution is crucial for transplanting quality assessment, site-specific seedling-stage management, and precision agricultural operations. However, seedlings in UAV orthomosaics are small, closely spaced, and easily confused with soil, shallow water, plastic mulch, and shadows, which makes accurate localization challenging. Meanwhile, under high-density planting, existing segmentation-based networks often generate adhesive responses between neighboring seedlings, leading to missed detections and biased counting. To address these issues, this study proposed PPNet, a deep learning framework for rice seedling localization and distribution mapping from UAV orthomosaics. The framework uses root-based point annotation and integrates a rice-point perception module (RPPM), an adjacent decoupling module (ADM), and an aggregated attention module (AAM). These modules enhance seedling-point responses, separate adjacent seedlings, and introduce row-structure information. Experiments were conducted on a UAV orthomosaic dataset containing 3,013 image tiles and 220,553 seedling point annotations from different rice-growing regions. PPNet achieved an F1 score of 0.9029, an AP of 0.8881, a counting MAE of 6.99, and an R2 of 0.9553. The method also generated field-scale seedling point maps and density maps under different planting densities and field backgrounds. The results demonstrate that PPNet can provide reliable seedling localization and counting information for crop establishment assessment and targeted seedling management, supporting intelligent agricultural machinery decision-making in precision farming operations.

## 1 Introduction

Rice is one of the most important food crops worldwide, and its production is closely related to food security (Peng et al., 2009; Bin Rahman and Zhang, 2023). Mechanical transplanting can improve transplanting efficiency; however, missing hills may still occur after transplanting because of seedling quality, transplanter operating conditions, and field conditions, thereby affecting rice yield. Therefore, accurate information on seedling number and location is important for evaluating transplanting quality and identifying locations requiring replanting (Li et al., 2024; Yeh et al., 2024).

Traditional rice seedling detection mainly relies on manual sampling. These methods can provide seedling counts for local areas, but they are labor-intensive and inefficient and cannot readily provide individual seedling locations across an entire field (Farjon et al., 2023). UAVs can rapidly acquire high-resolution imagery at the field scale. Before canopy closure, individual seedlings and planting rows remain distinguishable in orthomosaics, making UAV imagery suitable for rice seedling detection and counting (Zhang and Kovacs, 2012; Maes and Steppe, 2019). However, rice seedlings occupy only small regions in UAV images, and complex field backgrounds can also reduce recognition accuracy. Therefore, accurate recognition and localization of individual rice seedlings remain an important problem in UAV-based seedling monitoring.

Existing studies on seedling recognition have commonly used object detection and semantic segmentation. Object detection treats each seedling as an individual target and can directly provide its location and count (Yin et al., 2026). Tseng et al. (2022) used EfficientDet and Faster R-CNN to detect rice seedlings in UAV images; Anandakrishnan et al. (2025) proposed Li-YOLOv9 for rice seedling detection from large-scale UAV remote-sensing imagery; and Gao et al. (2025) developed PCERT-DETR for detecting rice seedlings and missing-seedling targets. Object detection can directly provide individual plant locations, but it requires a bounding box for each seedling. Because seedlings are small and their leaves have irregular shapes, individual plant boundaries are difficult to determine accurately under dense planting and leaf overlap, which also increases annotation effort. Semantic segmentation, in contrast, extracts seedling regions through pixel-wise classification. Zhang et al. (2023) proposed LW-SegNet and LW-UNet for segmenting UAV multispectral rice images containing different cultivars and planting densities; Liao et al. (2024) developed SC-Net for segmenting rice seedlings and weeds in paddy fields; and Yang et al. (2025) developed the HRNet-based RSHRNet for UAV rice seedling segmentation and mechanical transplanting quality assessment. These methods can extract seedling regions at the pixel level, but they do not directly distinguish individual plants within the same foreground region. When leaves of neighboring seedlings touch or overlap, the location of each individual seedling must still be further determined from the segmentation result.

In contrast, point annotation requires only the location of each seedling and is therefore more suitable than contour-based plant segmentation for counting and individual localization of dense small targets (Shan et al., 2026). Liang et al. (2022) addressed sparsely annotated semantic segmentation, in which only a small proportion of pixels are annotated using point-, scribble-, or block-wise supervision. They proposed Tree Energy Loss, which represents an image as a minimum spanning tree to model low-level and high-level pair-wise affinities and generates soft pseudo labels for unlabeled pixels in a coarse-to-fine manner. The loss can be combined with a conventional segmentation loss and incorporated into existing segmentation frameworks. Raine et al. (2022) addressed the high cost of pixel-level annotation in cluttered, multi-species coral reef imagery by propagating sparse point labels provided by ecologists within superpixel regions and subsequently training DeepLabV3+ for multi-species segmentation. In agricultural applications, Tong et al. (2021) proposed a point-wise supervised segmentation network that uses only tree-location points to train an encoder–decoder network and simultaneously predicts tree locations and individual masks for tree counting in UAV imagery. Zheng et al. (2023) proposed MLAENet to address scale variation, occlusion, and background interference in maize tassel counting. The network uses multiscale feature extraction and attention enhancement to generate point-supervised density maps and was evaluated on both fixed-camera and UAV imagery. Ruan et al. (2024) developed LettuceNet for high-density lettuce with small plant spacing, severe leaf overlap, and unclear boundaries between neighboring plants. The method uses only point-level labels for localization and counting, while LC-Loss encourages separate response regions for adjacent plants. These studies show that point supervision can achieve good recognition and localization performance while reducing annotation effort. However, shallow-water reflection, plastic mulch, shadows, and densely planted rows in paddy fields still pose difficulties for individual rice seedling localization.

To address these problems, this study proposes PPNet. Root-base point annotations are used to construct ground-truth maps, and RPPM, ADM, and AAM are incorporated to enhance seedling-point responses, separate neighboring seedlings, and exploit planting-row information. The predictions are further used to obtain individual seedling locations and counts and to generate field-scale seedling distribution maps.

The main contributions of this study are as follows:

1. A seedling-stage UAV orthomosaic dataset was constructed across multiple rice-producing regions and field conditions, containing 3,013 image tiles and 220,553 seedling point annotations.
2. PPNet, a rice seedling localization framework for UAV orthomosaics, was proposed to achieve individual seedling localization, seedling counting, and field-scale distribution mapping.
3. RPPM, ADM, and AAM were designed to enhance seedling-point responses, separate neighboring seedlings, and represent planting-row information, respectively.

## 2 Materials and methods

### 2.1 Overall framework

The overall workflow is shown in **Figure 1** and includes three steps. First, UAV images were collected in Heilongjiang, Anhui, and Guangdong, and field orthomosaics were generated using DJI Terra. The orthomosaics were tiled using a unified rule, and seedling root-base locations were annotated to build the training and validation datasets. Second, PPNet was developed by integrating RPPM, ADM, and AAM to enhance seedling-point responses, separate neighboring seedlings, and introduce row-structure context. Third, predicted seedling points were extracted from whole-field orthomosaics to calculate seedling counts and generate field-scale distribution maps.

**Figure 1:**
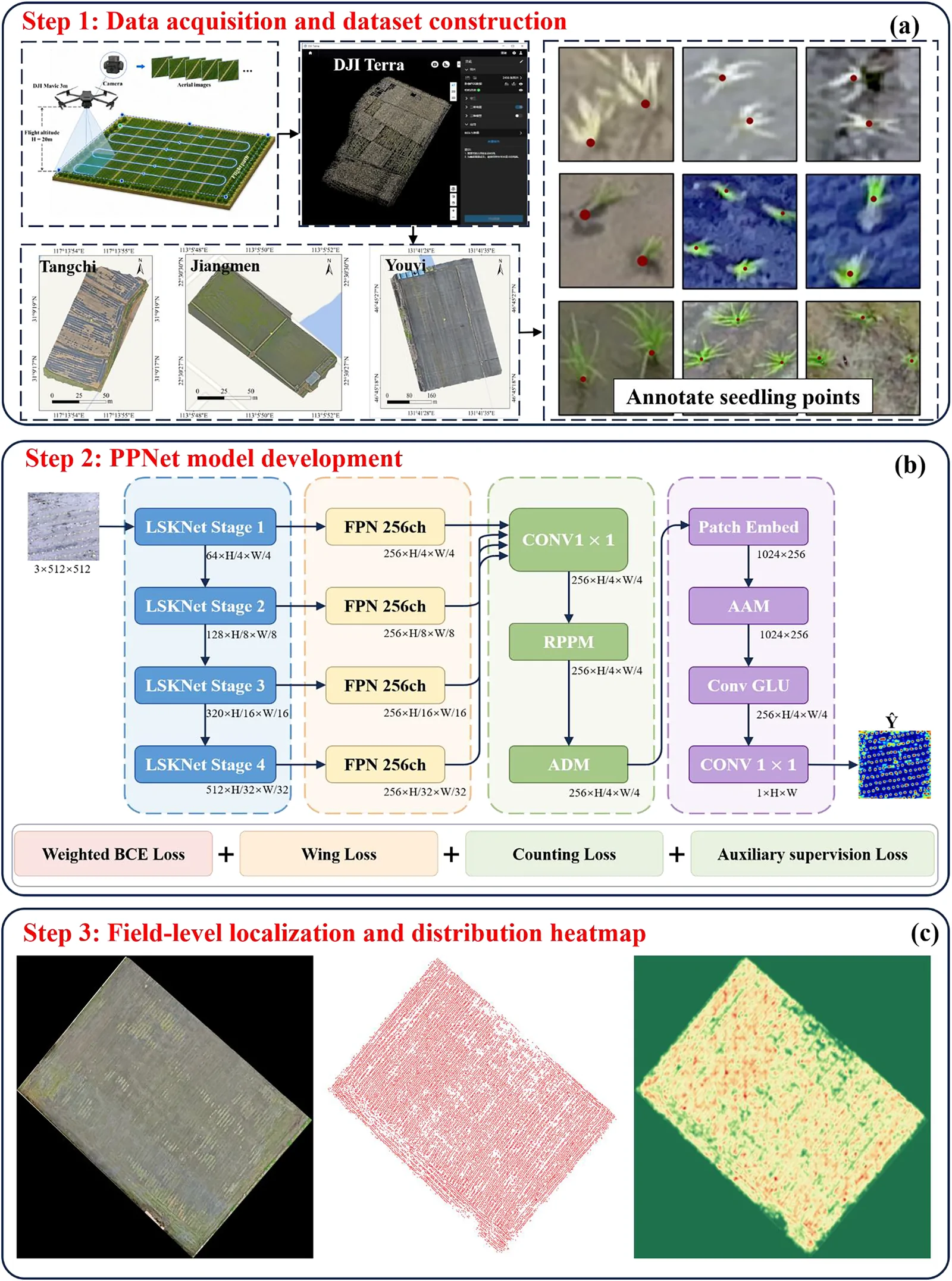
Overall framework of the proposed method. **(a)** Data acquisition and dataset construction. **(b)** PPNet model development. **(c)** Field-level seedling localization and distribution mapping.

### 2.2 Data acquisition and dataset construction

#### 2.2.1 Data acquisition

UAV image acquisition was conducted on April 18, 2025, in Jiangmen, May 15, 2025, in Tangchi, and May 24, 2025, in Youyi. The rice cultivars grown at the three sites were Jiangnong Xiangzhan 1, Zhongzao 39, and Longjing 31, respectively (**Figure 1a**). The sites differed in planting density, soil background, and mulching practice, providing diverse field conditions for model development and evaluation. Detailed information on the number of fields, within-row spacing, row spacing, flight missions, RGB images, and coverage area at each site is summarized in **Table 1**.

**Table 1:** Summary of field configuration and UAV image acquisition at the three study sites.

Study site	Fields	Within-row spacing (cm)	Row spacing(cm)	Flight missions	RGB images	Area (m²)
Youyi	6	13	30	2	2,118	78,220
Tangchi	4	18	24	1	428	5,564
Jiangmen	9	18	24	4	987	21,707
Total	19	—	—	7	3,533	105,491

Image acquisition was conducted from post-transplanting to early tillering. During this period, canopy closure had not yet occurred, and individual rice plants remained visually distinguishable in the aerial imagery. RGB images were acquired using a DJI Mavic 3 Multispectral (Mavic 3M, DJI, China) UAV equipped with an RTK positioning module and a 20-MP visible-light camera. The flight altitude was approximately 20 m at all sites, corresponding to a ground sampling distance (GSD) of approximately 0.5 cm/pixel. The flight routes were configured with forward and side overlaps of 80% and 75%, respectively, and a nominal flight speed of 5 m/s. RTK positioning was configured before flight operations to improve image georeferencing and subsequent orthomosaic reconstruction. The acquired RGB images were processed using photogrammetric reconstruction to generate georeferenced field-scale orthomosaics, which were subsequently used for rice seedling annotation, dataset construction, and field-scale seedling distribution analysis.

#### 2.2.2 Dataset construction

At the seedling stage, annotation based on canopy outlines or plant boundaries is easily affected by leaf overlap, lodging, and viewing angle, which may cause a mismatch between annotated centers and actual plant positions. In this study, each seedling was annotated at the root base so that the labeled point corresponded to the actual planting position. The orthomosaics (**Figure 2a**) were divided into 512 × 512-pixel image tiles with a stride of 512 pixels (**Figure 2b**), and seedling points were labeled using LabelMe. Two agricultural-domain specialists manually annotated the seedling points, and the annotations made by each specialist were independently checked by the other to identify and resolve missing, duplicated, or misplaced points.

**Figure 2:**
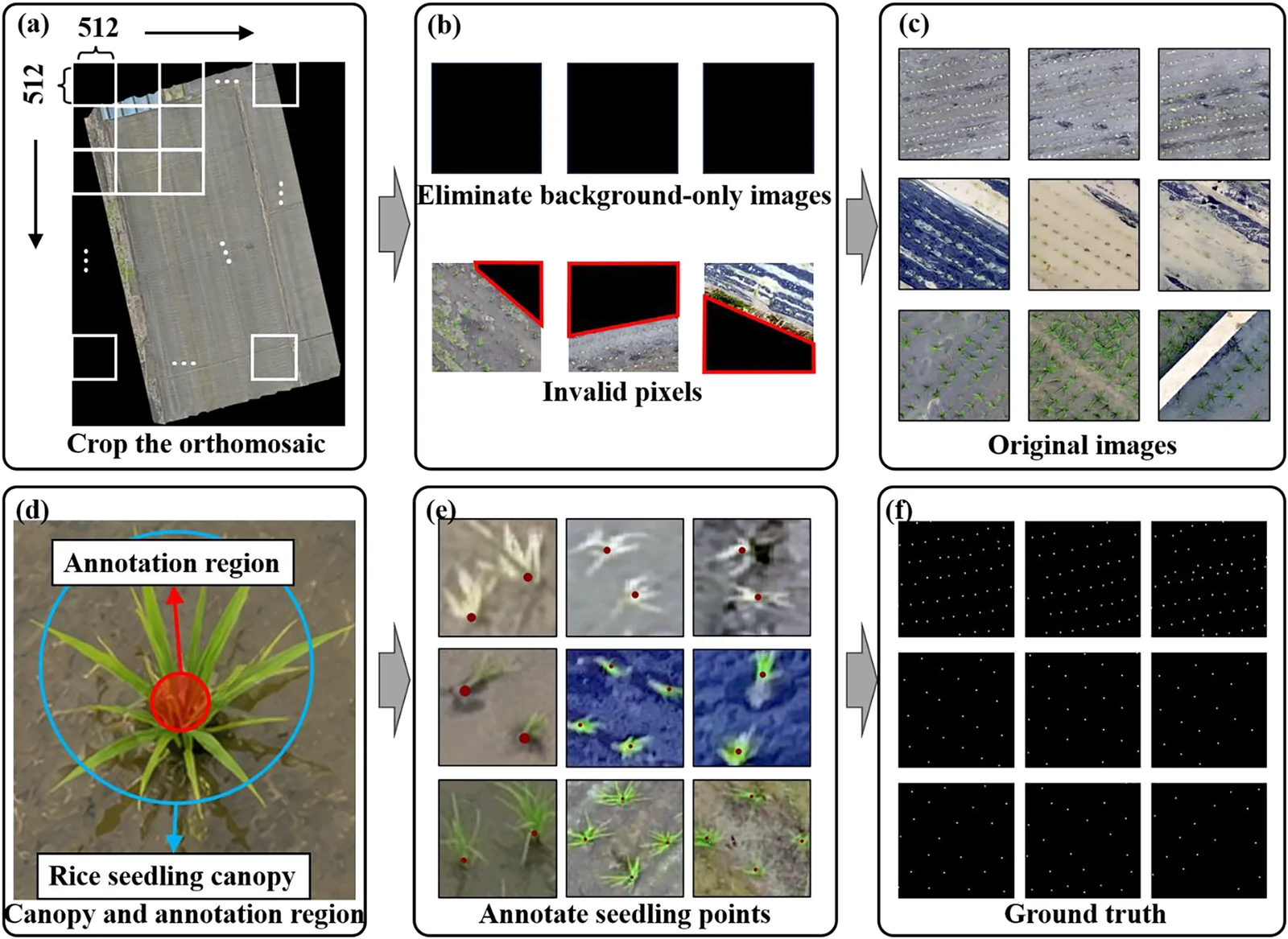
Dataset construction and rice seedling point annotation. **(a)** Field orthomosaics tiled into 512×512-pixel blocks. **(b)** Image blocks with invalid regions masked. **(c)** Images under different field conditions. **(d)** Root-based seedling point annotation. **(e)** Seedling point annotation results. **(f)** Ground-truth maps for model training generated from seedling points.

For challenging samples, annotation and verification followed unified root-base rules. Four typical difficult cases were handled as follows. (i) Water-surface reflection: when specular highlights obscured leaf contours but the planting hole or stem base remained visible, the annotation point was placed at the root base; if only reflected canopy fragments were visible and the root position could not be confirmed, the candidate was left unmarked and rechecked jointly by both annotators. (ii) Plastic-mulch occlusion: seedlings emerging from mulch holes were annotated at the root base within the planting hole rather than at the geometric center of the leaf canopy; seedlings completely covered by mulch with no visible stem or hole were not annotated. (iii) Seedling lodging: for lodged plants, the annotation was still placed at the root–soil interface rather than at the displaced canopy tip, so that the labeled point corresponded to the actual planting location. (iv) Shadow interference: when shadows reduced local contrast, annotators relied on stem continuity and planting-row context to locate the root base.

Image tiles containing only black background were excluded from model training (**Figure 2c**). Ground-truth maps were generated as circular regions with a diameter of 5 pixels centered on each verified annotation point (**Figures 2d–f**). Given a nominal ground sampling distance of approximately 0.5 cm/pixel, the 5-pixel diameter corresponds to approximately 2.5 cm on the ground, which is consistent with the centimeter-level positioning accuracy required for high-precision agricultural machinery guidance. The same annotation and quality-control procedures were applied to all three sites to reduce differences among annotators and regions.

Samples from each region were randomly divided into training and validation subsets, and the statistics are listed in **Table 2**. The dataset contains 3,013 image tiles and 220,553 seedling points, including 2,410 tiles and 177,091 points for training and 603 tiles and 43,462 points for validation. The average count was 73.2 seedlings per tile. Seedling density varied markedly among regions: Youyi averaged 120 points per tile, while Tangchi and Jiangmen averaged approximately 40 and 43 points per tile, respectively. Both the training and validation sets included samples from all three sites to reduce the influence of regional differences on model evaluation.

**Table 2:** Dataset statistics by region and split.

Region	Split	Images	Annotations	Avg./image
Youyi	Train	976	116,908	119.8
	Val	244	28,216	115.6
	Total	1,220	145,124	119.0
Tangchi	Train	317	12,803	40.4
	Val	72	2,820	39.2
	Total	389	15,623	40.2
Jiangmen	Train	1,117	47,380	42.4
	Val	287	12,426	43.3
	Total	1,404	59,806	42.6
All	Train	2,410	177,091	73.5
	Val	603	43,462	72.1
	Total	3,013	220,553	73.2

### 2.3 Problem formulation

For the k−th UAV orthomosaic image $I_{k}$, the ground-truth seedling point set is defined as $G_{k}={\left\{g_{k,n}\right\}}_{n=1}^{N_{k}}$, where $N_{k}$ is the true seedling count in that image. A ground-truth map $Y_{k}$ is constructed by placing a circular region of 5-pixel diameter centered at each seedling point. PPNet learns the mapping from the input image to the predicted map, as shown in Equation 1.

(1) $$f_{\theta }:I_{k}\mapsto {\hat{Y}}_{k}$$

where θ denotes the network parameters. Predicted seedling points are extracted from ${\hat{Y}}_{k}$ to form the set defined in Equation 2.

(2) $${\hat{P}}_{k}={\left\{{\hat{p}}_{k,m}\right\}}_{m=1}^{{\hat{N}}_{k}}$$

where ${\hat{N}}_{k}=|{\hat{P}}_{k}|$ is the predicted seedling count. The objective is to reduce discrepancies between predicted and ground-truth seedling points in both location and number under complex field backgrounds.

### 2.4 PPNet network architecture

#### 2.4.1 Overall architecture

The architecture of PPNet is shown in **Figure 1b**. UAV orthomosaic tiles are first input into a four-stage LSKNet backbone to extract multi-level features, which are then fused by a feature pyramid network (FPN). The fused features are processed by RPPM, ADM, and AAM in sequence. RPPM strengthens seedling-point features, ADM separates neighboring seedlings under dense planting, and AAM aggregates local and row-structure information. The network finally outputs a seedling probability map with the same spatial resolution as the input image.

#### 2.4.2 Rice-point perception module

RPPM (**Figure 3**) takes the FPN-fused neck feature as input, denoted by F. During training, ground-truth maps are formed by circular regions of 5-pixel diameter centered at each seedling point. Conventional 3×3 and 5×5 square convolutions sample unevenly in different directions and do not match the circular shape of the supervision regions. RPPM applies ring-shaped masks to convolution kernels on F and fuses multi-radius branch feature maps to strengthen feature expression at seedling-point locations.

**Figure 3:**
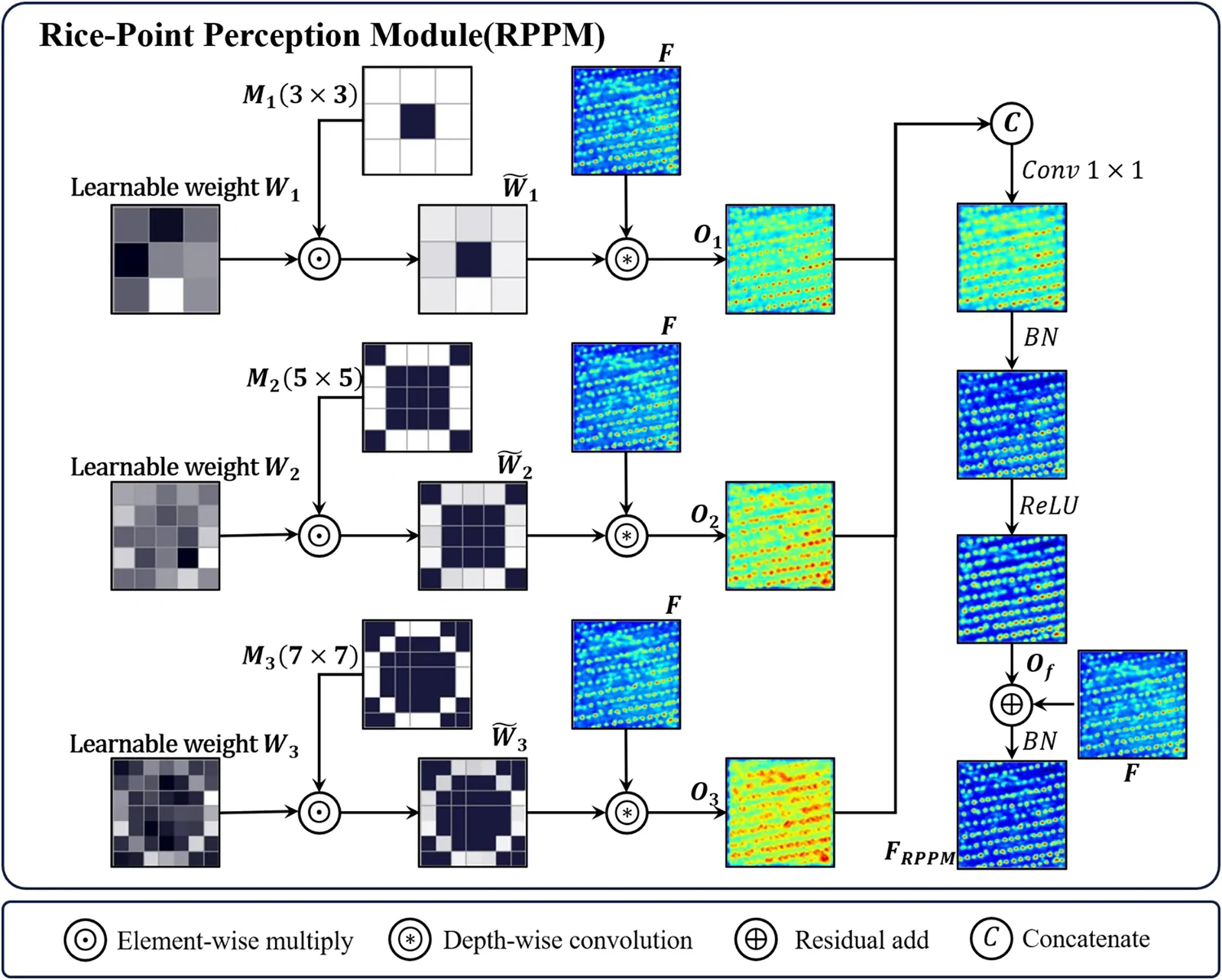
Structure of the Rice-Point Perception Module.

Let the radius set be R={1,2,3}, corresponding to 3×3, 5×5, and 7×7 receptive fields, respectively. For each r∈R, a binary ring mask $M_{r}$ is constructed in a (2r+1)×(2r+1) window with center (r,r). Let $d_{ij}$ be the Euclidean distance from pixel (i,j) to the center; $M_{r}(i,j)=1$ if $r-\frac{1}{2}\leq d_{ij}\leq r+\frac{1}{2}$, and 0 otherwise.

Each radius branch uses depthwise separable convolution with learnable kernel weights $W_{r}$. The effective kernel is ${\tilde{W}}_{r}=W_{r}⊙M_{r}$, and the branch output is calculated using Equation 3.

(3) $$O_{r}=\text{DSConv}(F;{\tilde{W}}_{r})$$

Outputs ${\left\{O_{r}\right\}}_{r\in R}$ are concatenated along the channel dimension, passed through a 1×1 convolution, batch normalization, and a nonlinear activation, and projected back to the same channel dimension as F to obtain $O_{f}$. A residual connection is used to obtain the RPPM output, as shown in Equation 4:

(4) $$F_{\text{RPPM}}=\text{BN}(O_{f}+F)$$

This design enhances seedling-point responses while preserving spatial information in the neck features.

#### 2.4.3 Adjacent decoupling module

Although RPPM strengthens seedling-point responses, neighboring seedlings may still exhibit merged activations on the feature map under dense planting, affecting subsequent point extraction. ADM (**Figure 4**) models central and larger surrounding neighborhoods on $F_{\text{RPPM}}$ and uses differencing to reduce mutual interference between adjacent plants.

**Figure 4:**
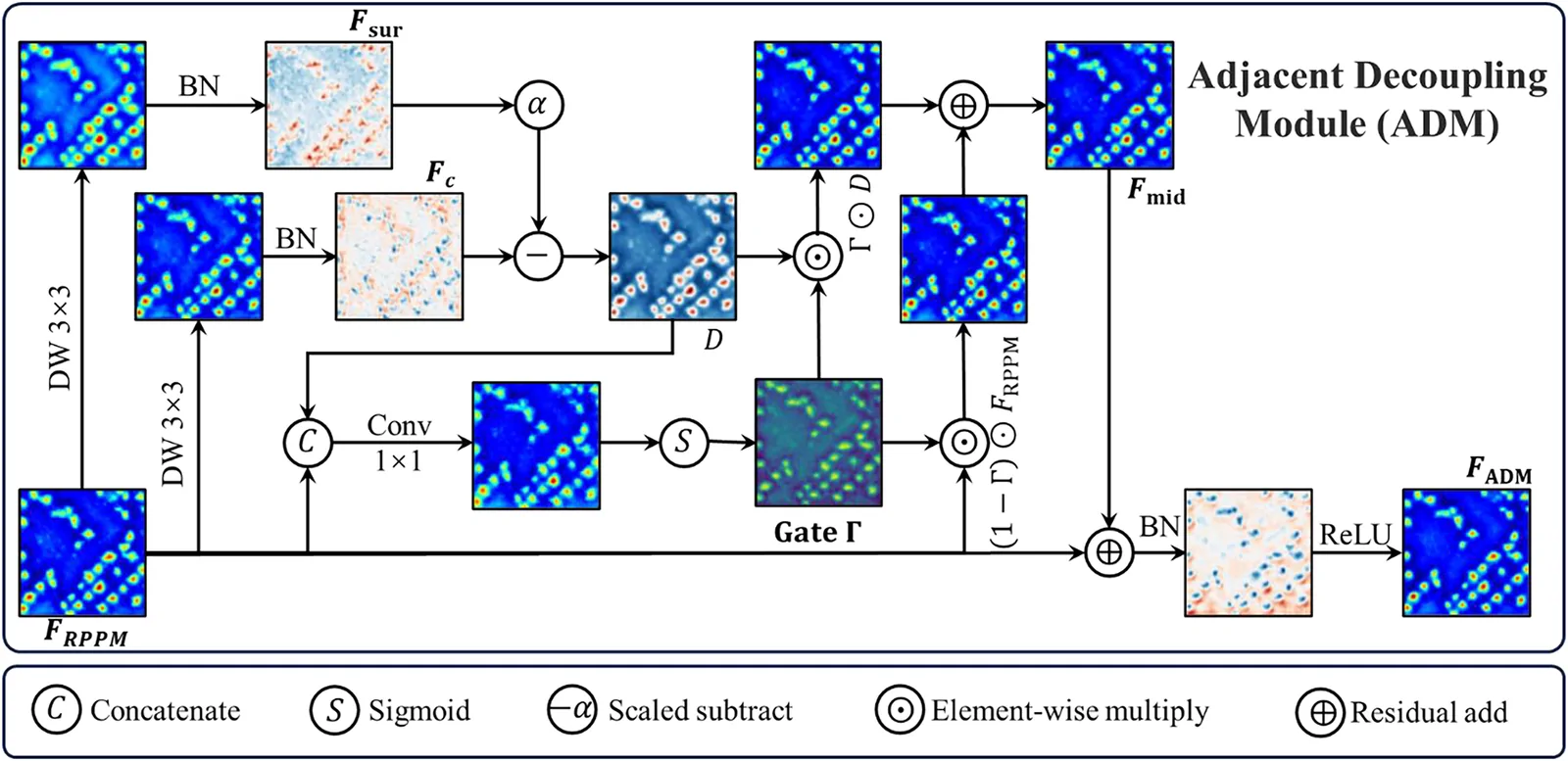
Structure of the Adjacent Decoupling Module.

The center branch uses a 3×3 depthwise separable convolution with batch normalization to produce $F_{\text{C}}$; the surround branch uses a dilated 3×3 depthwise separable convolution with batch normalization to produce $F_{sur}$. A learnable coefficient α constrained to [0.1,0.9] controls the suppression strength of surround features, and the difference feature is defined in Equation 5.

(5) $$D=F_{c}-\alpha F_{\text{sur}}$$

Using D alone may over-suppress useful features in low-density seedling areas or simple-background regions. Therefore, $F_{\text{RPPM}}$ and D are concatenated along the channel dimension, passed through a 1×1 convolution and Sigmoid to obtain gating weights Γ, and *F*_mid_ is computed as shown in Equation 6:

(6) $$F_{\text{mid}}=\Gamma ⊙D+(1-\Gamma )⊙F_{\text{RPPM}}$$

After batch normalization and ReLU, a residual connection with $F_{\text{RPPM}}$ yields the ADM output, as shown in Equation 7.

(7) $$F_{\text{ADM}}=\text{ReLU}(\text{BN}(F_{\text{mid}}+F_{\text{RPPM}}))$$

ADM mitigates response merging between adjacent seedlings in dense regions while preserving original information, providing a clearer feature basis for subsequent prediction.

#### 2.4.4 Aggregated attention module

Although $F_{\text{ADM}}$ already separates many neighboring seedlings, seedling localization also depends on local texture and row structure at a larger scale. Local convolutions alone cannot capture long-range spatial relations; global self-attention on high-resolution feature maps is computationally expensive and may weaken fine local differences in dense regions. AAM (**Figure 5**) combines local-window and downsampled regional context on $F_{\text{ADM}}$ and outputs a seedling response map at the same resolution as the input image.

**Figure 5:**
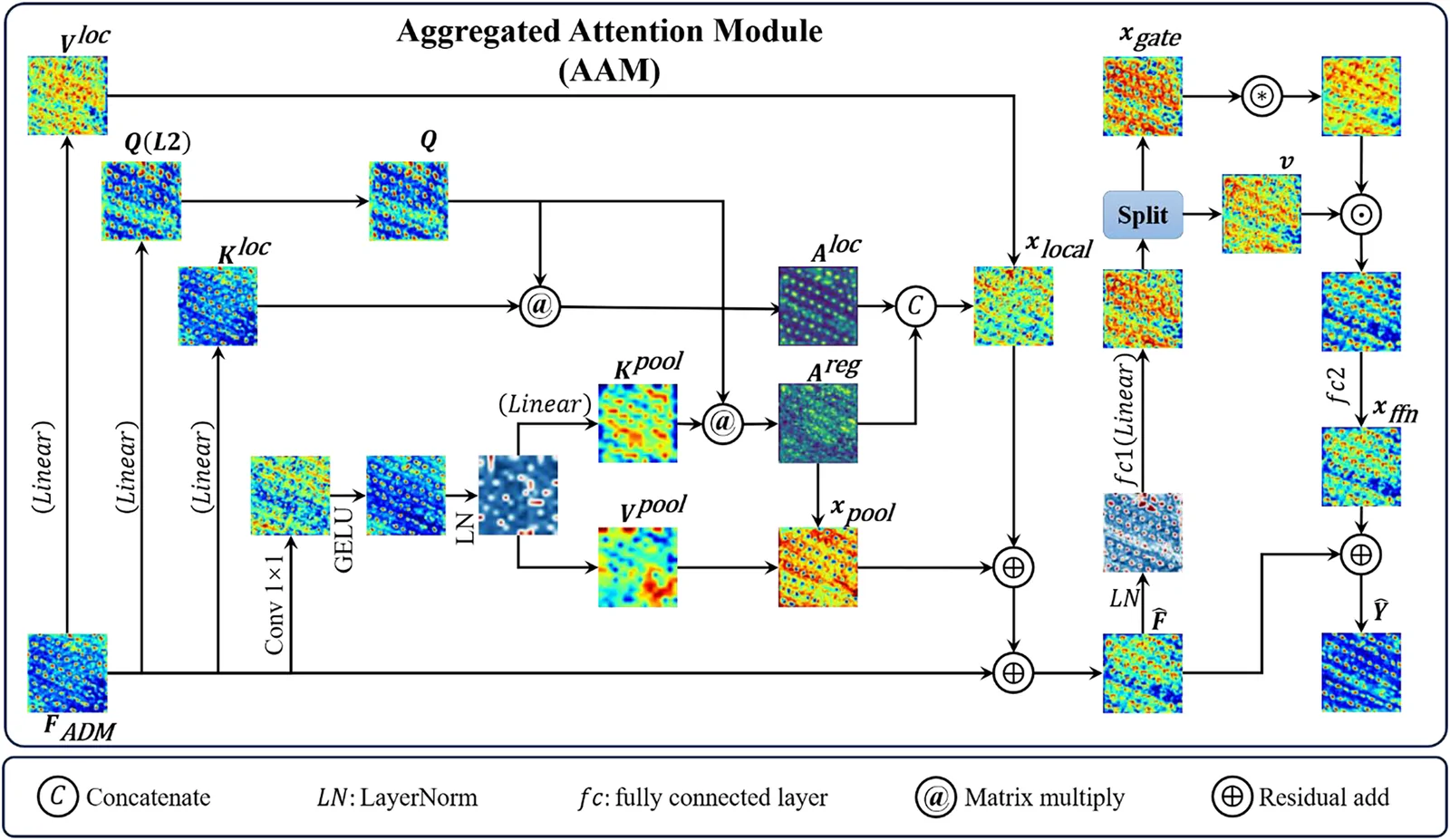
Structure of the Aggregated Attention Module.

The aggregated attention sublayer models two types of spatial context through local and regional branches. After layer normalization of $F_{\text{ADM}}$, linear projections produce L2-normalized queries Q. The local branch projects keys $K^{loc}$ and values $V^{loc}$ from the same input and unfolds local windows via an unfold operation. The regional branch downsamples the feature map and projects L2-normalized keys $K^{pool}$ and values $V^{pool}$. The local and regional attention scores are calculated using Equation 8.

(8) $$A^{\text{loc}}=Q^{\left(K^{\text{loc}}\right)}+b^{\text{loc}},A^{\text{reg}}=Q^{\left(K^{\text{pool}}\right)}+b^{\text{reg}}$$

where $b^{\text{loc}}$ is a learnable relative position bias and $b^{\text{reg}}$ is generated by an MLP from continuous relative coordinates. The two score tensors are concatenated and normalized by a unified Softmax, split according to local and regional lengths, and used to weight $V^{loc}$ and $V^{pool}$. The weighted features are summed, linearly projected, and added residually to obtain intermediate features $\hat{F}$, as shown in Equation 9.

(9) $$\hat{F}=F_{\text{ADM}}+\text{proj}(x_{\text{local}}+x_{\text{pool}})$$

A convolutional gated feed-forward sublayer applies layer normalization to $\hat{F}$, expands features linearly, and splits them into two paths, $x_{\text{gate}}$ and v, as defined in Equation 10.

(10) $$\left[x_{\text{gate}},v]=\text{split}({\text{fc}}_{1}(\text{LN}(\hat{F}))\right)$$

A 3×3 depthwise convolution introduces local spatial structure into $x_{\text{gate}}$, which is activated by GELU and multiplied element-wise with v, then projected back to the original dimension using Equation 11.

(11) $$x_{\text{ffn}}={\text{fc}}_{2}(\text{GELU}(\text{DWConv}(x_{\text{gate}}))⊙v)$$

The two sublayers are applied sequentially with residual connections to obtain the block output $F^{\left(l\right)}$, as expressed in Equation 12.

(12) $$F^{\left(l\right)}=\hat{F}+x_{\text{ffn}}$$

After $N_{\text{blk}}=6$ identical blocks, the feature map is upsampled to the original resolution and passed through a 1×1 convolution to produce the prediction map according to Equation 13.

(13) $$\hat{Y}={\text{Conv}}_{1\times 1}(\text{Upsample}(\text{LN}(F^{\left(N_{\text{blk}}\right)}))),\hat{Y}\in {\mathbb{R}}^{B\times 1\times H\times W}$$

After AAM processing, the network preserves local seedling responses while incorporating remote row-structure information along seedling rows.

#### 2.4.5 Loss function

For the k−th image, the main branch outputs a prediction map ${\hat{Y}}_{k}$ and the ground-truth map is $Y_{k}$. The normalized prediction is defined in Equation 14.

(14) $$S_{k}=\sigma ({\hat{Y}}_{k})$$

where σ(·) is the Sigmoid function. Invalid pixels at orthomosaic tile boundaries are excluded from loss computation; the set of valid pixels is denoted $\Omega_{k}$. The per-image loss comprises four terms: main-branch supervision, shape constraint, counting constraint, and auxiliary supervision, as detailed below.

(1) Weighted BCE loss

Weighted binary cross-entropy is computed on logits ${\hat{Y}}_{k}$ and $Y_{k}$ to alleviate extreme foreground–background imbalance, as shown in Equation 15.

(15) $$L_{\text{BCE}}=-\frac{1}{|\Omega_{k}|}\sum_{(i,j)\in \Omega_{k}}\left[w_{+}Y_{k,ij}\text{ln}\sigma ({\hat{Y}}_{k,ij})+w_{-}(1-Y_{k,ij})\text{ln}(1-\sigma ({\hat{Y}}_{k,ij}))\right]$$

where $w_{+}=\rho$, $w_{-}=1$, and ρ=17.2 in this study. The weighting coefficient in the total loss is $\lambda_{1}=4.0$.

(2) Wing loss

To constrain the response shape near seedling points, Wing loss is applied to the absolute error between the normalized prediction and the ground-truth map on valid pixels. The pixel-wise Wing loss is defined in Equation 16. The mean Wing loss over all valid pixels is then calculated using Equation 17.

(16) $$l_{\text{wing}}(u)=\{\begin{array}{cc} \omega \text{ln}(1+u/\epsilon ), & u<\omega ,\\ u-\omega +\omega \text{ln}(1+\omega /\epsilon ), & u\geq \omega , \end{array}$$

(17) $$L_{\text{wing}}=\frac{1}{\left|\Omega_{k}\right|}\sum_{(i,j)\in \Omega_{k}}l_{\text{wing}}(u_{ij})$$

(3) Counting loss

To align the overall response level of the prediction map with the ground-truth map, the responses on valid pixels are summed and compared in logarithmic form using the counting loss defined in Equation 18.

(18) $$L_{\text{cnt}}=|\ln(\sum_{(i,j)\in \Omega_{k}}S_{k,ij}+1)-\ln(\sum_{(i,j)\in \Omega_{k}}Y_{k,ij}+1)|$$

This term acts on continuous response maps during training; at test time, discrete point sets ${\hat{P}}_{k}$ and counts ${\hat{N}}_{k}$ are obtained from $S_{k}$ via post-processing.

(4) Auxiliary supervision loss

A shallow auxiliary branch outputs ${\hat{Y}}_{k}^{\text{aux}}$ , and the corresponding auxiliary loss is defined in Equation 19.

(19) $$L_{BCE}^{aux}=-\frac{1}{|\Omega_{k}|}\sum_{(i,j)\in \Omega_{k}}\left[w_{+}Y_{k,ij}\text{ln}\sigma {(}_{{\hat{Y}}_{k,ij}}^{aux})+w_{-}(1-Y_{k,ij})\text{ln}(1-\sigma {(}_{{\hat{Y}}_{k,ij}}^{aux}))\right]$$

The total loss for the k−th image is defined in Equation 20.

(20) $$L_{k}=\lambda_{1}L_{\text{BCE}}+\lambda_{2}L_{\text{wing}}+\lambda_{3}L_{\text{cnt}}+\lambda_{\text{aux}}L_{\text{BCE}}^{\text{aux}}$$

The weighting coefficients were set to $\lambda_{1}=4.0$, $\lambda_{2}=0.5$, $\lambda_{3}=0.1$, and $\lambda_{\text{aux}}=0.4$ based on preliminary experiments on the validation set and were kept fixed for all experiments.

### 2.5 Training settings

Experiments were conducted on Ubuntu 20.04 using an NVIDIA GeForce RTX 4090 GPU (24 GB memory) with PyTorch 2.0.1 and CUDA 11.8. LSKNet was used as the backbone. The input image size was 512×512 pixels, the batch size was 8, and training ran for 44,200 iterations. Optimization used AdamW with an initial learning rate of 6×10^-5^, a weight decay of 0.05, and $\beta_{1}=0.9$, $\beta_{2}=0.999$. The learning rate was decayed following a poly schedule with linear warmup in the early training stage. Gradient clipping was applied with a maximum norm of 0.1.

### 2.6 Metrics for localization and counting

For the *k*-th image, the network output ${\hat{Y}}_{k}$ is converted to a normalized prediction map $S_{k}=\sigma ({\hat{Y}}_{k})$, $S_{k}\in {\left[0,1\right]}^{H\times W}$. After Gaussian smoothing of $S_{k}$, local maxima are extracted and filtered by non-maximum suppression and threshold τ to obtain the predicted seedling point set ${\overset{⌢}{P}}_{k}$; the ground-truth point set $G_{k}$ is obtained from manual annotation. The predicted and true seedling counts are defined in Equation 21.

(21) $${\hat{N}}_{k}=|{\hat{P}}_{k}|,N_{k}=|G_{k}|,k=1,\ldots ,K$$

Candidate matches between ${\overset{⌢}{P}}_{k}$ and $G_{k}$ are established based on Euclidean distance, and one-to-one optimal matching is performed using the Hungarian algorithm. Matches with distance no greater than the tolerance radius $d_{th}$ are counted as true positives (TP); unmatched predictions are false positives (FP) and unmatched ground-truth points are false negatives (FN). After aggregating TP, FP, and FN over the validation set, precision, recall, and F1 are calculated using Equation 22.

(22) $$\text{Precision}=\frac{\text{TP}}{\text{TP}+\text{FP}},\text{Recall}=\frac{\text{TP}}{\text{TP}+\text{FN}},F_{1}=\frac{2\cdot \text{Precision}\cdot \text{Recall}}{\text{Precision}+\text{Recall}}$$

F1 at a fixed threshold τ is used as the primary localization metric. Precision–recall curves are obtained by varying τ, and average precision is defined in Equation 23.

(23) $$\text{AP}=\int_{0}^{1}P(R)dR$$

which measures the overall matching quality between predicted and ground-truth seedling points across thresholds.

The mean absolute error is defined in Equation 24.

(24) $$\text{MAE}=\frac{1}{K}\sum_{k=1}^{K}|{\hat{N}}_{k}-N_{k}|$$

The coefficient of determination is defined in Equation 25.

(25) $$R^{2}=1-\frac{\sum_{k=1}^{K}{\left({\hat{N}}_{k}-N_{k}\right)}^{2}}{\sum_{k=1}^{K}{\left(N_{k}-\bar{N}\right)}^{2}},\bar{N}=\frac{1}{K}\sum_{k=1}^{K}N_{k}$$

which quantify the agreement between predicted and true seedling counts.

## 3 Results and analysis

### 3.1 Localization performance comparison

Seedling localization in UAV orthomosaics is often implemented by extracting plant positions from dense prediction maps. Therefore, PPNet was compared with five representative semantic segmentation networks widely used in plant phenotyping and agricultural image analysis: DeepLabV3+, HRNet, SCTNet, SegFormer, and UNet. All methods used the same training-validation split, input resolution, optimization settings, and training iterations. During inference, seedling points were extracted from the output maps using the same post-processing strategy, and Precision, Recall, F1, and AP were calculated. The quantitative results are summarized in **Table 3**.

**Table 3:** Comparison of seedling point localization performance among different methods.

Method	Precision	Recall	*F1*	*AP*
DeepLabV3+	0.6011	0.5236	0.5597	0.3718
HRNet	0.8623	0.7713	0.8142	0.6887
UNet	0.8652	0.8187	0.8413	0.7672
SegFormer	0.8934	0.7763	0.8308	0.6926
SCTNet	0.8744	0.7908	0.8305	0.6896
PPNet (Ours)	**0.8668**	**0.9422**	**0.9029**	**0.8881**

Bold values indicate the best-performing result for each evaluation metric.

PPNet achieved the best localization performance, with an F1 score of 0.9029 and an AP of 0.8881. Compared with the strongest baseline, UNet, PPNet improved F1 and AP by about 6.1 and 12.1 percentage points, respectively, and obtained the highest recall of 0.9422. The difference is mainly related to the characteristics of rice seedlings in UAV orthomosaics. Seedlings are small, closely spaced, and often mixed with exposed soil, shallow water, plastic mulch, and shadows. DeepLabV3+ performed much worse than the other methods, indicating that its coarse semantic features were insufficient for preserving the fine spatial details required for seedling localization. HRNet, SegFormer, SCTNet, and UNet all achieved F1 values above 0.81, showing the value of high-resolution features and multi-scale information. However, their recall values remained clearly lower than that of PPNet, indicating that neighboring seedlings were still merged or missed under dense planting. PPNet maintained both high precision and high recall, suggesting that it better preserved individual seedling locations in complex field conditions. Its AP of 0.8881 further indicates stable localization quality across confidence thresholds, which is important for subsequent counting and field-scale mapping.

Qualitative comparisons in **Figure 6** further show the differences among the methods under complex field conditions. In the shallow-water scene, water reflection and exposed wet soil caused obvious omissions in DeepLabV3+ and HRNet, while SegFormer also missed several seedlings in the highlighted area. UNet and SCTNet retained more seedling locations, but local discontinuities were still present. PPNet produced a more complete distribution and preserved the planting pattern more consistently. On plastic mulch, the bright background and local reflectance around planting holes reduced the contrast of small seedlings. DeepLabV3+ and HRNet showed more missed detections, whereas SegFormer and SCTNet produced local breaks along the planting rows. UNet performed better in this scene but still missed several seedlings in the highlighted region. Under shadowed dense planting, the differences among the methods became more pronounced. DeepLabV3+, HRNet, and SegFormer showed obvious omissions, and some neighboring seedlings were represented by incomplete or merged predictions. SCTNet and UNet recovered more seedlings but did not fully preserve the dense within-row distribution. In comparison, PPNet retained more individual seedling locations and showed better agreement with the ground truth. These results show that PPNet preserves a more complete seedling distribution under water reflection, plastic mulch, and shadowed dense-planting conditions.

**Figure 6:**
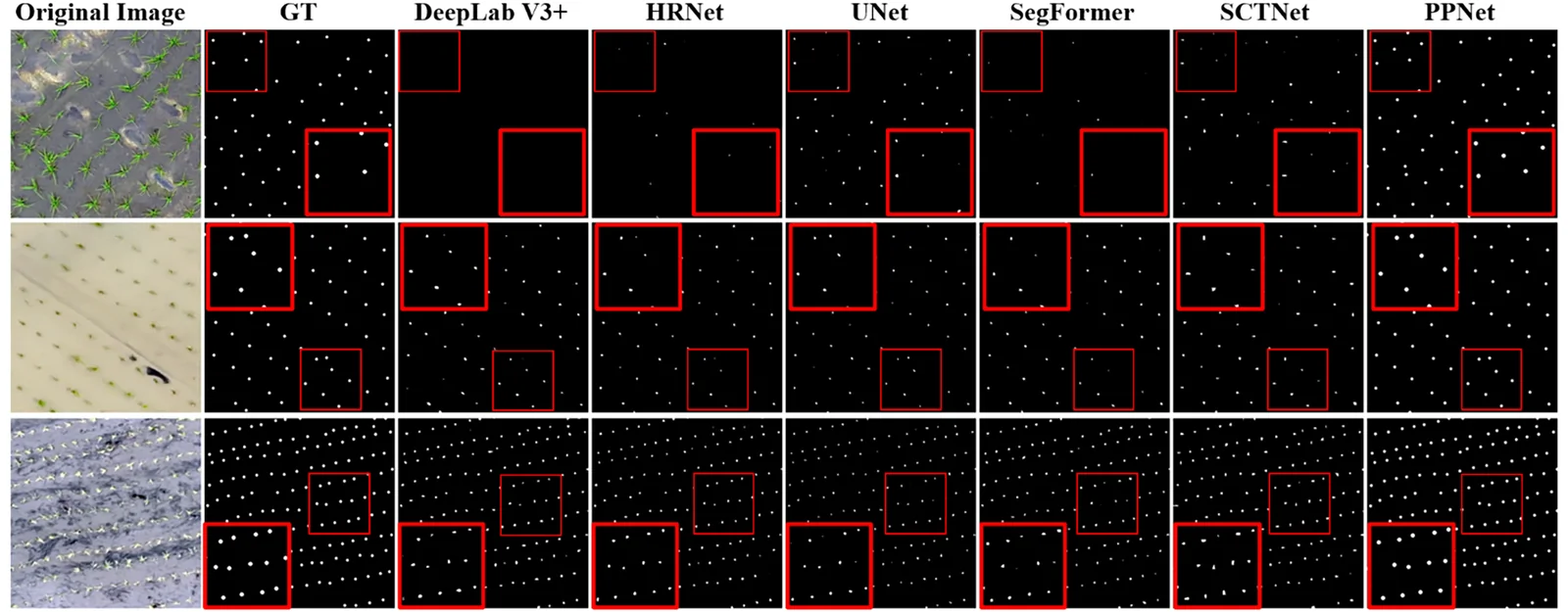
Visual comparison of rice seedling localization under different field conditions. The three rows represent shallow-water, plastic-mulch, and shadowed dense-planting scenes, respectively. Red boxes indicate representative challenging areas.

To further quantify point-level detection behavior, confusion matrices were constructed using the seedling point matching results (**Figure 7**). PPNet achieved the highest TP count (40,950) and the lowest FN count (2,512) among all methods. DeepLabV3+ had an FN count of 20,705, close to its TP count of 22,757, indicating that nearly half of the actual seedlings were missed. HRNet, SegFormer, and SCTNet showed FN counts from 9,092 to 9,940, while UNet reduced the FN count to 7,880.

**Figure 7:**
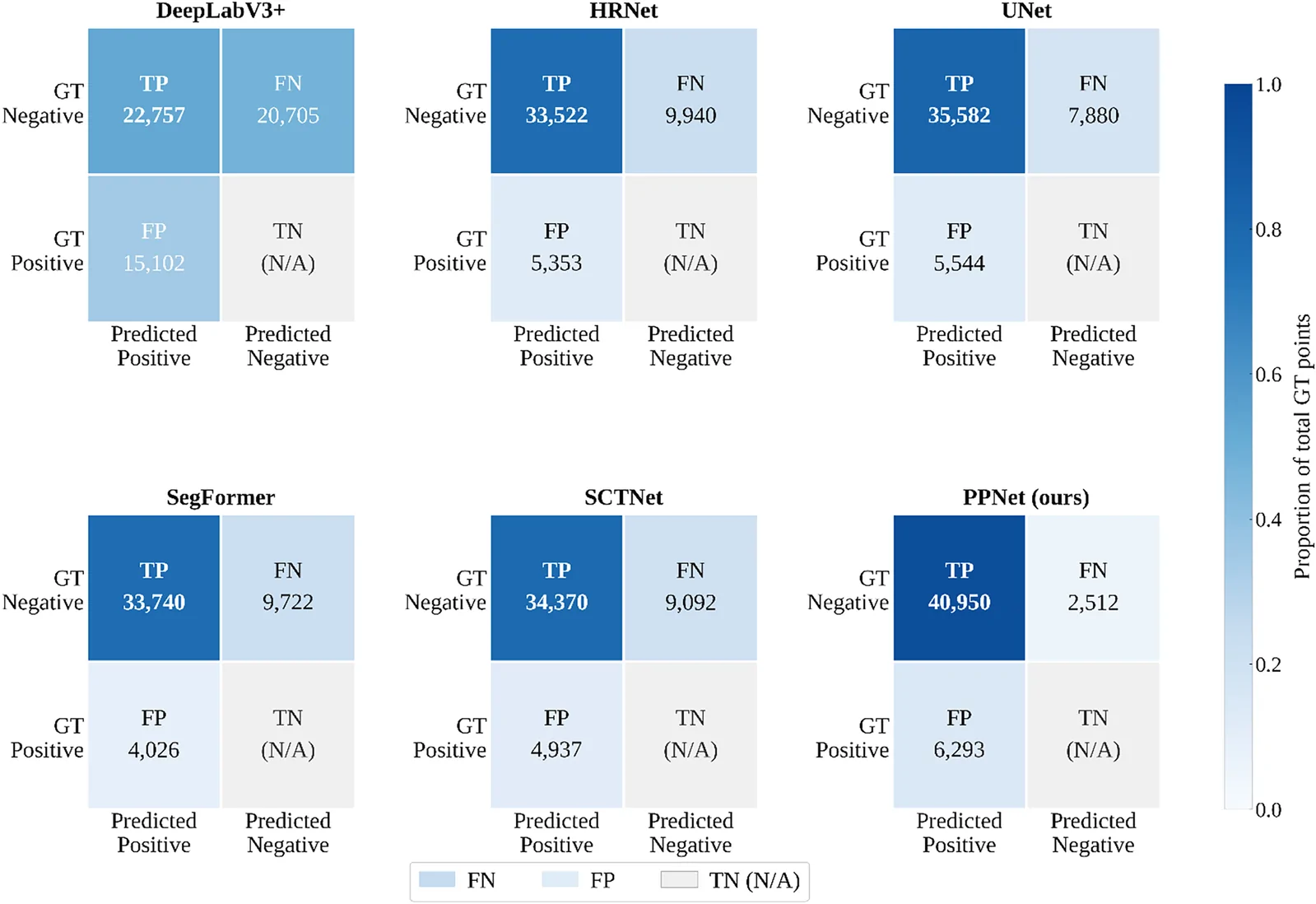
Point-level detection confusion matrices of all compared methods on the validation set (total GT seedling points: 43,462). TP, correctly detected seedlings; FN, missed seedlings; FP, false alarms. TN is not applicable because detection performance is evaluated through point-to-point matching, in which only TP, FP, and FN are defined.

The TP-FN-FP distribution reflects different failure modes. SegFormer had the lowest FP count (4,026) but a relatively high FN count (9,722), indicating a conservative detection tendency. DeepLabV3+ produced the highest FP count (15,102), suggesting weak background discrimination in complex field environments. HRNet showed high counts in both FN (9,940) and FP (5,353), reflecting limitations in seedling separation and background suppression. PPNet reduced FN substantially while maintaining a moderate FP count (6,293), which is consistent with its slight tendency to overestimate seedling counts.

The high FN counts of the comparison methods were mainly caused by response merging under dense planting. When adjacent seedlings produce overlapping activation regions, several plants may be matched to only one prediction point and the remaining seedlings are counted as missed detections. PPNet alleviates this problem through the center-surround suppression mechanism in ADM, which helps maintain independent response peaks for neighboring seedlings. From an application perspective, the TP difference between PPNet (40,950) and DeepLabV3+ (22,757) corresponds to about 18,000 additional detected seedlings across the validation set. In field-scale missing-plant assessment, such omissions would cause many low-density areas to be overlooked and would reduce the reliability of replanting and zonal management decisions.

### 3.2 Computational cost and inference speed

SegFormer had the fewest parameters (3.71 M), with 6.77 G FLOPs and 357.2 FPS. SCTNet required only 4.71 G FLOPs and achieved the highest FPS (578.6). DeepLabV3+ required 26.43 G FLOPs but still reached 518.8 FPS. HRNet had 9.64 M parameters, 18.66 G FLOPs, and 129.2 FPS. UNet had 24.89 M parameters and 225.84 G FLOPs, indicating a much higher computational cost than most baselines.

PPNet had 23.62 M parameters, close to UNet, but its FLOPs were 60.56 G, much lower than those of UNet. Its FPS was 62.0, lower than most lightweight baselines. Although this speed is acceptable for offline orthomosaic analysis, it remains insufficient for real-time onboard perception on intelligent agricultural machinery. This limitation mainly arises from the additional computation introduced by RPPM, ADM, and AAM. Future work will explore model pruning, knowledge distillation, and lighter attention designs to reduce computational cost while preserving localization accuracy. Overall, PPNet trades part of the inference speed for improved localization and counting accuracy, which is acceptable when field-scale accuracy is the main requirement.

### 3.3 Component ablation analysis

To evaluate the contribution of each component, an ablation study was conducted while keeping the backbone, feature fusion strategy, loss functions, and training settings unchanged. Four configurations were tested. Configuration A used the backbone, FPN, and an FCN decoder without RPPM, ADM, or AAM. Configuration B added RPPM to the baseline. Configuration C further added ADM. Configuration D was the complete PPNet, in which the FCN decoder was replaced by AAM. All configurations were evaluated on the same validation dataset using the same localization metrics. The results are summarized in **Table 4**.

**Table 4:** Ablation results for different module combinations.

Configuration	RPPM	ADM	AAM	Precision	Recall	*F1*	*AP*
A	✗	✗	✗	0.8132	0.9425	0.8731	0.8872
B	✓	✗	✗	0.8264	**0.9477**	0.8829	0.8850
C	✓	✓	✗	0.8287	0.9612	0.8900	0.8876
D	✓	✓	✓	**0.8668**	0.9422	**0.9029**	**0.8881**

Bold values indicate the best-performing result for each evaluation metric.

The ablation results indicate that the three modules influence different types of localization errors rather than yielding uniform improvements across all metrics. Configuration A achieved a high recall of 0.9425 but a substantially lower precision of 0.8132, suggesting that false-positive responses constituted a major source of error in the baseline model. After RPPM was introduced in configuration B, precision increased to 0.8264 and recall increased to 0.9477. Based on the 43,462 ground-truth seedling points in the validation set, these changes correspond to approximately 226 fewer missed seedlings and 757 fewer false-positive predictions. However, AP decreased from 0.8872 to 0.8850, indicating that the improvement associated with RPPM was primarily observed at the operating threshold used for point extraction rather than across the full precision–recall curve. After ADM was further introduced in configuration C, recall increased from 0.9477 to 0.9612, whereas precision changed only slightly from 0.8264 to 0.8287. This corresponds to approximately 587 additional correctly localized seedlings, while the number of false-positive predictions remained nearly unchanged. The primary effect of ADM was therefore a reduction in missed detections rather than suppression of false responses. This observation is consistent with the response maps in **Figures 8** and **9**, in which previously merged responses between neighboring seedlings become more clearly separated after ADM is introduced.

**Figure 8:**
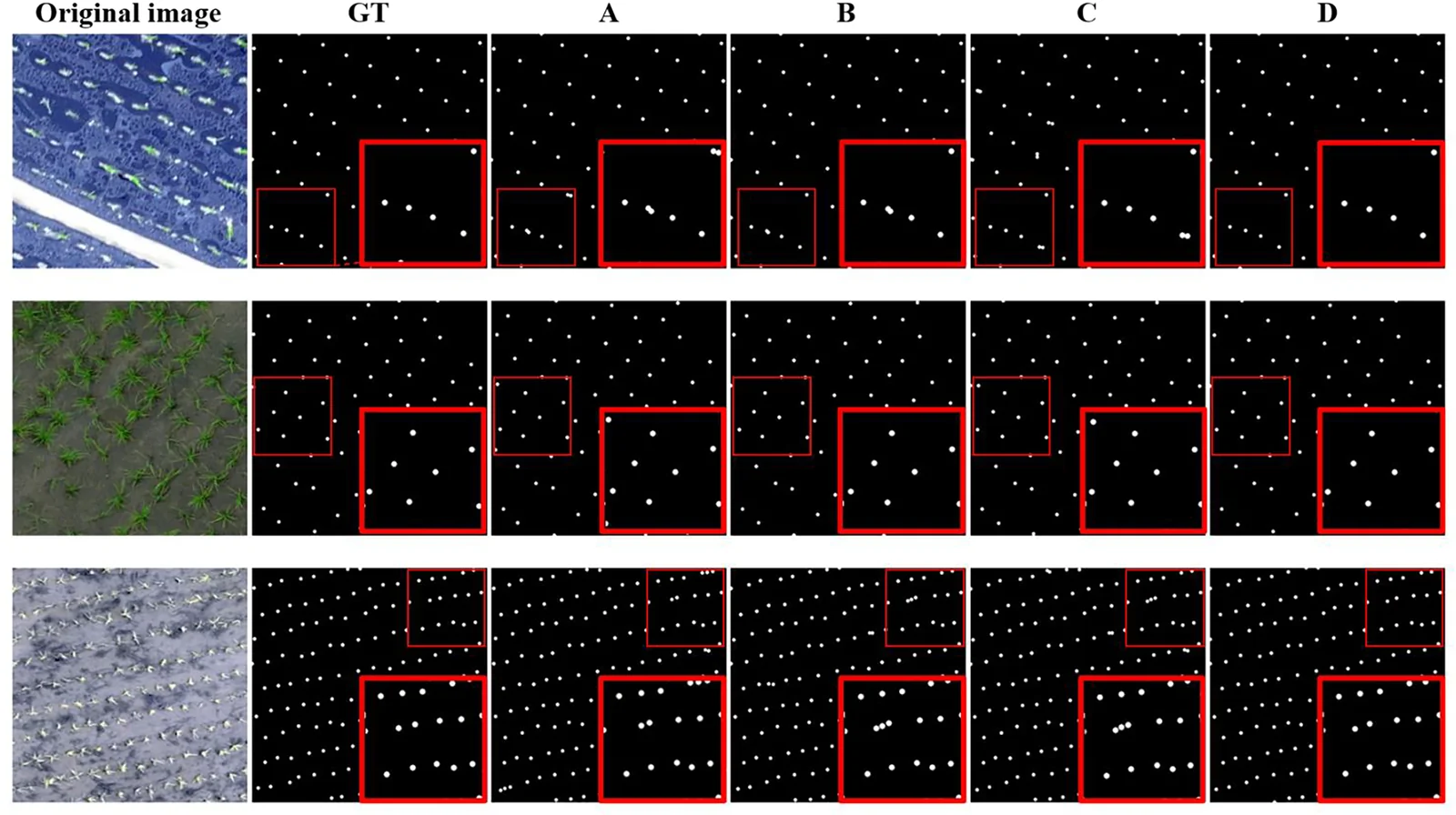
Visual comparison of seedling point localization results in the ablation study.

**Figure 9:**
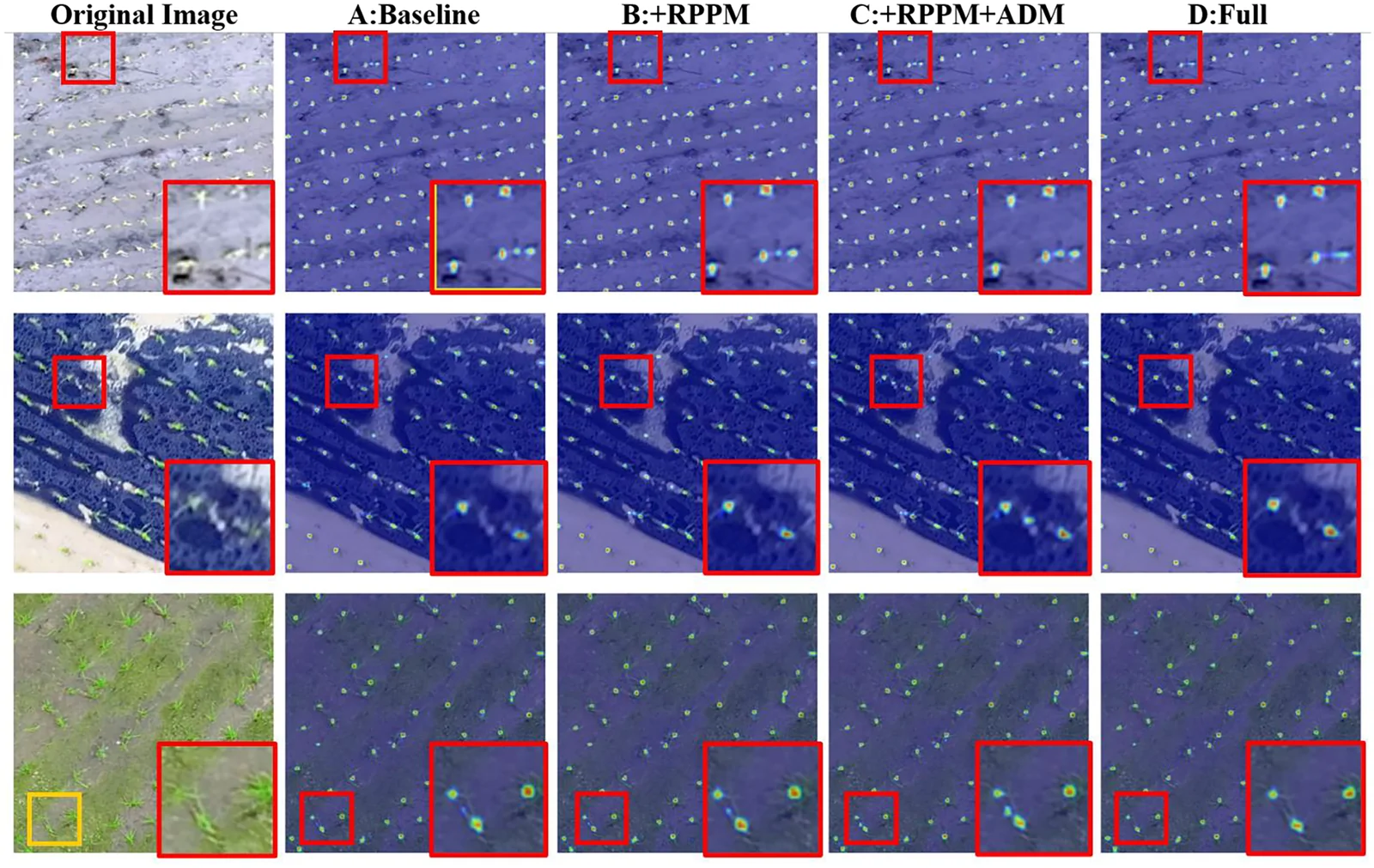
Comparison of seedling response maps generated by different ablation configurations.

Introducing AAM in configuration D produced a different redistribution of localization errors. Precision increased markedly from 0.8287 to 0.8668, whereas recall decreased from 0.9612 to 0.9422. Based on the 43,462 ground-truth seedling points in the validation set, these changes correspond to approximately 2,343 fewer false-positive predictions and approximately 826 additional missed seedlings. This trade-off is consistent with the design of AAM. By aggregating local-window responses with downsampled row-structure context, AAM strengthens regular within-row patterns and suppresses scattered activations that are inconsistent with planting geometry. Such suppression effectively reduces background false positives caused by soil texture, water reflection, mulch edges, and shadows. At the same time, seedlings with weak appearance cues—such as partially occluded, lodged, or low-contrast plants near tile boundaries—may produce only marginal responses. When these weak peaks deviate from the dominant row context, AAM can attenuate them together with background clutter, which explains the slight decline in recall. In other words, AAM improves localization reliability mainly by filtering geometrically inconsistent responses rather than by recovering additional seedlings. Combined with the preceding modules, the overall mechanism can be summarized as follows: RPPM enhances seedling-point responses and improves the initial discriminability of root-base locations; ADM separates merged activations between neighboring plants and thereby reduces missed detections under dense planting; and AAM introduces row-level structural priors that further suppress false positives, at the cost of suppressing a small number of marginal true seedlings. Consequently, the complete PPNet shifts from a high-recall but relatively noisy predictor toward a more precise localization model, which is reflected by the increase in F1 from 0.8900 to 0.9029.

Compared with configuration A, the complete PPNet maintained recall at a similar level (0.9425 vs. 0.9422), while precision increased by 0.0536 and F1 increased by 0.0298. The number of correctly localized seedlings was therefore largely preserved, whereas false-positive predictions were reduced by approximately one third. Overall, the complete model primarily reduced false-positive responses while maintaining the seedling recovery capability of the baseline. The limited change in AP from 0.8872 to 0.8881 should be interpreted in relation to the metric definitions and the performance of the baseline model. AP characterizes matching quality across the full range of confidence thresholds, whereas F1 is computed at the fixed operating threshold adopted for point extraction. The baseline configuration already achieved high recall (0.9425) and a relatively high AP (0.8872), indicating that the backbone and FPN were capable of recovering most seedling locations over a broad confidence range. Under these conditions, the dominant residual error was associated with false-positive responses rather than extensive missed detections across thresholds. RPPM, ADM, and AAM primarily refined response quality near the selected operating point: RPPM enhanced seedling-point responses, ADM separated neighboring seedlings in dense rows, and AAM suppressed scattered background responses by incorporating row-level context. Therefore, the ablation results demonstrate that the proposed modules are most effective for improving reliable seedling localization under the practical operating threshold, although the corresponding gain in AP is limited.

The qualitative results in **Figure 8** are consistent with the quantitative results. In the first-row sample, the baseline produced discontinuous row segments and several merged seedling locations. After RPPM was introduced, the seedling rows became more complete. ADM further separated neighboring seedlings within dense row segments. The complete PPNet generated seedling distributions most consistent with the ground-truth annotations. A similar pattern was observed in the second-row sample. The baseline and RPPM-only configurations missed several seedlings in the highlighted region, indicating that some plants were not well distinguished from the background. After ADM was added, most of these missed seedlings were recovered and the row structure became more complete. AAM further reduced irregular responses and produced a distribution closer to the actual planting pattern. The differences among configurations were more evident in the third-row sample, which contained dense planting and strong background interference. The baseline showed broken row structures and obvious response merging. As RPPM, ADM, and AAM were introduced, seedling rows became clearer and more continuous. The complete PPNet produced the most accurate within-row distribution and showed the strongest agreement with the ground-truth annotations.

The response maps in **Figure 9** further explain these differences. From Configuration A to D, seedling response regions became more compact and concentrated around actual seedling locations. RPPM enhanced responses near seedling centers, ADM separated merged responses in dense regions, and AAM suppressed scattered background responses by using row-level context. These effects jointly produced clearer seedling representations, stronger separation between neighboring plants, and reduced background interference.

### 3.4 Counting accuracy and field-scale distribution mapping

In addition to seedling localization, accurate estimation of seedling density and spatial distribution is important for evaluating crop establishment at the field scale. This section therefore analyzes counting performance under different planting densities and presents the field-scale seedling distribution maps generated by PPNet.

The influence of planting density on counting performance is shown in **Figure 10**. Under sparse planting, the counting differences among methods were small because neighboring seedlings were well separated. As planting density increased, the differences became clearer. In the medium-density sample, DeepLabV3+ and HRNet substantially underestimated the seedling count, and SCTNet, SegFormer, and UNet also produced lower counts than the ground truth. PPNet predicted 59 seedlings, close to the true count of 60. Under high-density conditions, neighboring seedlings were frequently clustered and the comparison methods showed stronger underestimation. PPNet predicted 146 seedlings, close to the true count of 145. These results indicate that dense planting is a major source of counting error in UAV orthomosaics. When adjacent seedlings generate overlapping responses, several plants may be represented by one predicted point. Therefore, counting accuracy depends not only on seedling recognition but also on the separation of neighboring plants. The stable performance of PPNet across density levels indicates its advantage in preserving individual seedling representations under dense planting.

**Figure 10:**
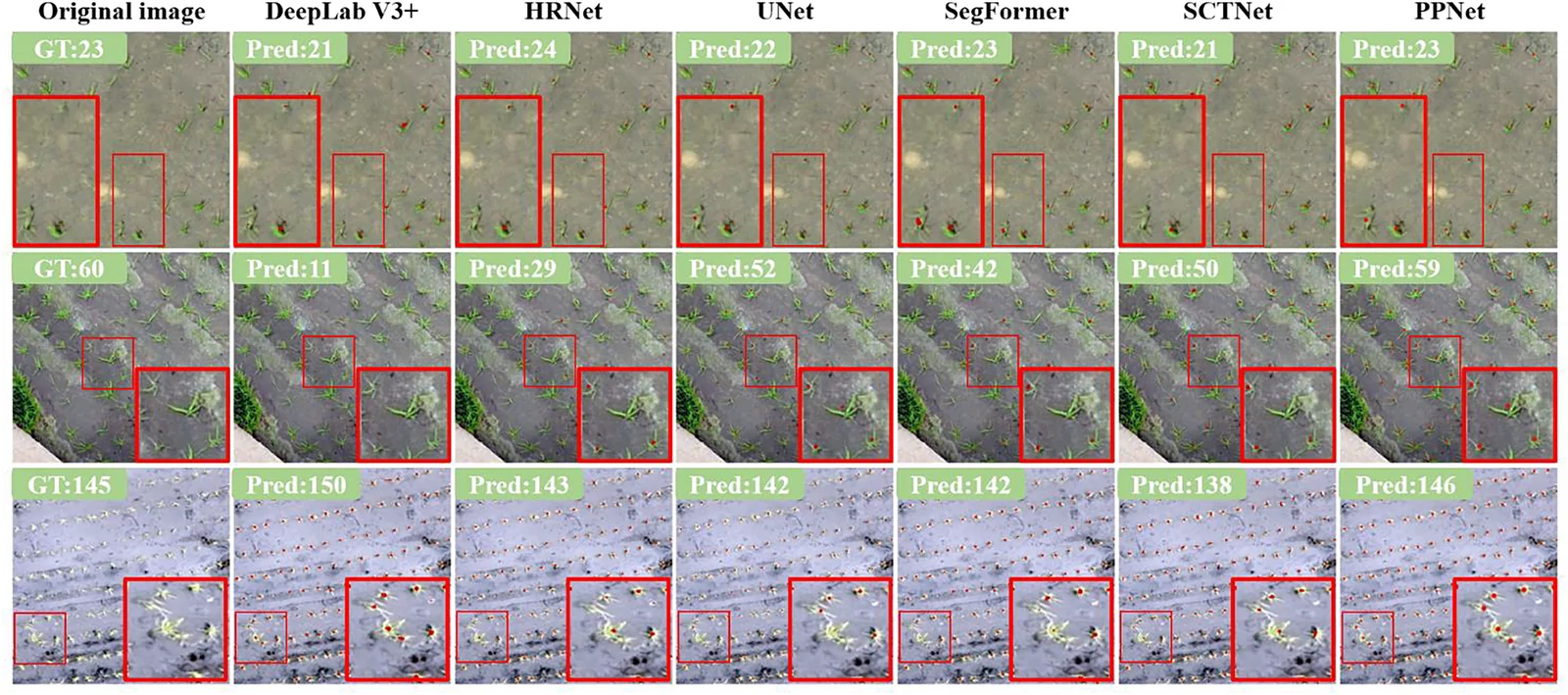
Comparison of localization results among different methods under varying planting densities.

PPNet can also be used to map rice seedling distribution across whole fields (**Figure 11**). The seedling point maps show clear row patterns that match the planting layout, and the density maps show where seedlings are concentrated or sparse. Among the three orthomosaics, the second field had a higher and more continuous seedling density, while the third field contained several low-density patches. These patterns are difficult to capture by traditional sampling because sampling covers only limited locations. UAV-based mapping provides a more complete view of seedling establishment and can help identify differences related to transplanting quality, field conditions, or early growth. The maps also have direct management value: low-density areas may indicate poor establishment or missed transplanting and can be targeted for replanting or other corrective measures, while high-density areas may require different water or nutrient management. By combining UAV imagery with seedling localization and counting, PPNet provides a practical way to assess field-scale establishment status and support site-specific management during the seedling stage. To further evaluate counting consistency across the validation dataset, the relationship between predicted and true seedling counts was analyzed.

**Figure 11:**
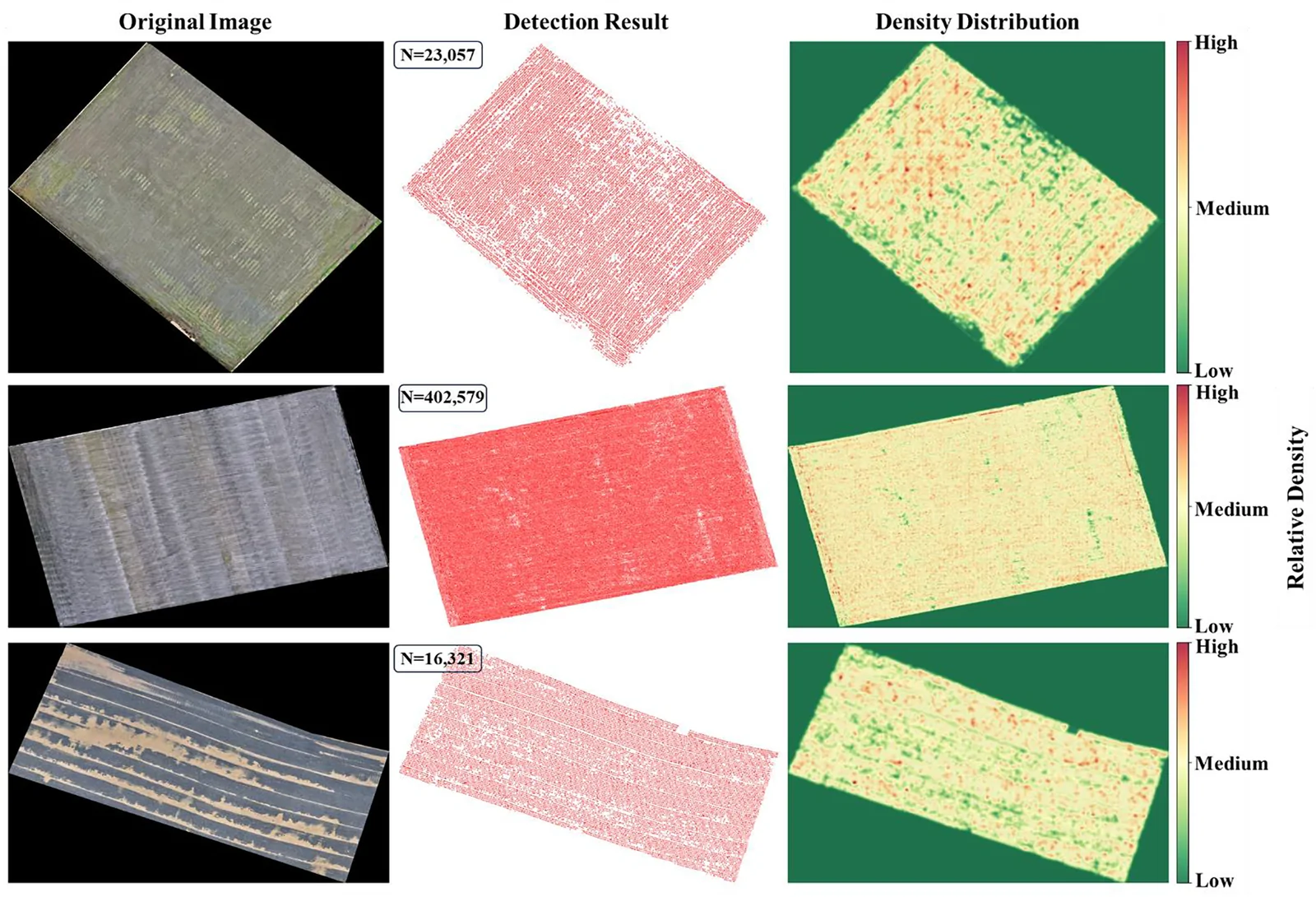
Field-scale rice seedling distribution.

**Figure 12** shows the relationship between predicted and true seedling counts for all evaluated methods. The scatter plots show a clear association between localization quality and counting accuracy. Methods with lower localization performance had larger deviations from the 1:1 reference line and higher counting errors. DeepLabV3+ and HRNet showed the largest scatter dispersion and the highest MAE values, indicating that localization errors were directly transferred to counting errors. SegFormer, SCTNet, and UNet produced more concentrated scatter distributions and higher coefficients of determination.

**Figure 12:**
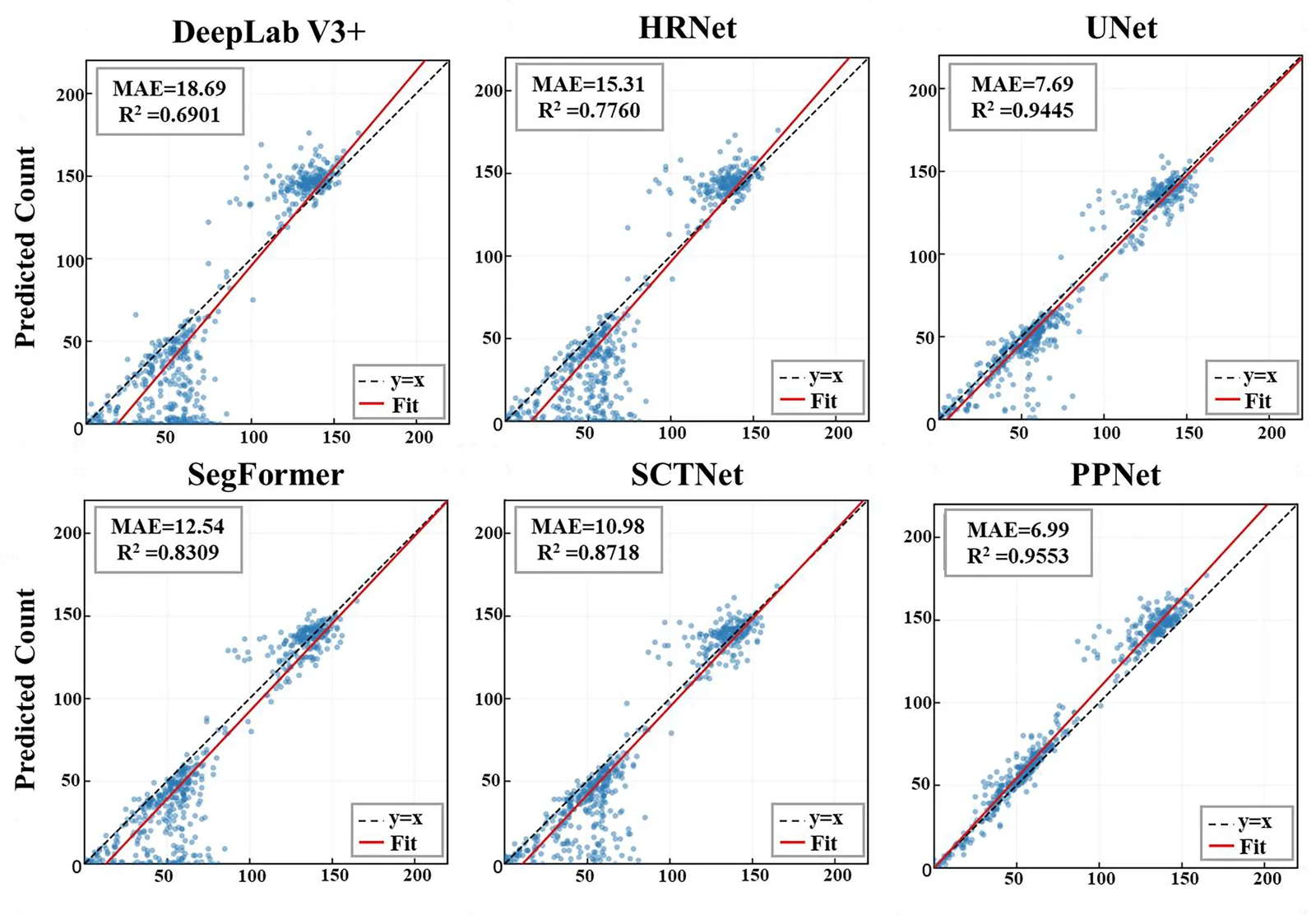
Scatter plots of predicted versus true seedling counts for different methods.

Among all methods, PPNet showed the strongest agreement between predicted and true seedling counts, with an MAE of 6.99 and an *R^2^* of 0.9553. Most samples were close to the 1:1 reference line, indicating stable counting performance over a wide range of seedling densities. Because seedling count is derived from detected seedling locations, missed detections and merged seedlings directly lead to count underestimation. The counting results therefore further confirm that PPNet can preserve individual seedling locations and reduce localization errors under complex field conditions.

## 4 Discussion

### 4.1 Point-based seedling localization with adjacent decoupling and row-structure guidance

Accurate identification of rice seedlings from UAV orthomosaics remains challenging because seedlings occupy only a small proportion of image pixels and often occur under dense planting, complex soil backgrounds, and variable illumination. These factors can cause adjacent seedlings to merge in prediction maps, resulting in missed plants and biased seedling counts. The problem is more evident at the early growth stage, when individual seedlings are small and neighboring plants are closely spaced.

Previous studies have shown that UAV imagery is effective for high-resolution crop monitoring and field-scale crop assessment, especially during early growth stages when canopy structures are still visible (Zhang and Kovacs, 2012; Yang et al., 2017; Maes and Steppe, 2019; Yang et al., 2020; Sun et al., 2022; Tao et al., 2022; Lyu et al., 2023). UAV-based methods have been applied to rice seedling detection, wheat seedling counting, and maize stand estimation because they can rapidly collect spatial information over large fields (Liu et al., 2022; Tseng et al., 2022; Zhang et al., 2023). However, reliable identification of individual plants remains difficult when seedlings are dense, small, and affected by background variation.

In this study, rice seedlings were represented by root-base point annotations rather than segmentation masks or bounding boxes. This strategy provides a direct representation of seedling positions and avoids uncertainty caused by leaf shape, overlap, and partial occlusion. It also reduces the inclusion of background pixels, which is important for small targets in UAV images. Similar conclusions have been reported in wheat seedling studies and dense object counting tasks (Song et al., 2021; Wang et al., 2024; Zang et al., 2025). The results of this study further demonstrate that root-based point annotation is suitable for rice seedling localization under different planting densities and field conditions.

The improved performance of PPNet is mainly related to its use of both local seedling features and row-level planting information. Mechanically transplanted rice fields usually have regular row structures, but dense planting reduces the visual separation between neighboring seedlings. PPNet enhances seedling-background discrimination, separates adjacent seedlings, and uses row-structure information to refine predictions. As a result, the predicted seedling locations are more consistent with actual crop rows across different regions and planting densities.

The results also indicate that high-resolution UAV orthomosaics contain sufficient spatial detail for seedling-stage rice assessment. When combined with point-based annotation and deep learning, UAV imagery can describe seedling distribution over large fields more completely than manual counting or quadrat sampling. This capability is useful for large-scale rice production, where seedling establishment often varies among fields and regions (Zhang and Kovacs, 2012; Yang et al., 2017; Maes and Steppe, 2019).

### 4.2 Field-scale seedling mapping for precision field operations

The seedling distribution maps and density maps generated by PPNet reflect differences in seedling emergence among different fields and management practices. From a field management perspective, these maps may help identify areas that warrant further inspection: low-density areas may indicate poor establishment or missed transplanting, and high-density areas may be associated with uneven emergence or localized management differences. By combining UAV observations with seedling localization and counting, PPNet provides a technically feasible approach for evaluating transplanting quality and supporting field-scale crop monitoring (Zhang and Kovacs, 2012; Yang et al., 2017; Maes and Steppe, 2019). Whether such maps can be translated into specific management actions, such as targeted replanting or variable-rate input application, remains to be validated in on-farm trials.

Several limitations should be noted. First, the framework was developed mainly for the seedling stage before canopy closure, when individual seedlings are still distinguishable in UAV imagery. As the crop grows, increasing leaf overlap may reduce the visibility of individual plants and consequently affect localization accuracy. Second, although this study demonstrates the technical feasibility of the proposed framework, further work is needed to integrate it with actual agricultural machinery control systems and validate its performance under real field operating conditions. Third, although the dataset covers multiple rice-growing regions and field conditions, the training and validation sets were derived from the same study regions. The resulting similarity in environmental background, cultivation practices, and imaging conditions may lead to an optimistic estimation of model performance and limit the assessment of true cross-region generalizability. Fourth, the loss weights were selected on the validation set, and the same set was used for final reporting. Future work could derive additional operational indicators, such as density uniformity, row continuity, and missing-plant patch distribution. Multi-temporal UAV observations could also be used to monitor crop development throughout the growing season. In addition, combining this framework with crop growth models or 3D structural traits may further support precision field management (Jin et al., 2020, Jin et al., 2021; Liu et al., 2021; Miao et al., 2021; Yang et al., 2024; Dong et al., 2025). Ultimately, the spatially resolved seedling distribution information provided by PPNet can serve as a data foundation for precision field management decisions, contributing to efficient, green, and low-cost rice production.

## 5 Conclusion

This study proposed PPNet, a deep learning framework for rice seedling localization and spatial distribution mapping from UAV orthomosaics. Using root-based seedling point annotations as supervision, PPNet integrates RPPM, ADM, and AAM to address challenges including small seedling size, dense planting arrangement, and complex field backgrounds. The framework completes seedling localization, counting, and field-scale distribution mapping in a unified workflow.

Experiments were conducted on a multi-regional UAV orthomosaic dataset containing 3,013 image tiles and 220,553 seedling point annotations. The results demonstrate that PPNet outperformed representative semantic segmentation methods in both localization and counting. The framework achieved an F1 score of 0.9029, an AP of 0.8881, a counting MAE of 6.99, and an R² of 0.9553. It also showed stable performance across different planting densities and field backgrounds on the validation samples from the three study sites.

PPNet further generated whole-field seedling distribution maps that revealed differences in seedling density and planting patterns within rice fields. These maps may provide useful information for evaluating transplanting quality and identifying areas with poor seedling establishment after field validation. Several limitations remain. The model is intended for the seedling stage before canopy closure. Evaluation was based on a random within-region tile split without an independent held-out field or year, so the reported metrics may be slightly optimistic. In addition, integration with agricultural machinery and on-farm validation of management decisions still require further work.

## Data Availability

The source code and representative sample images supporting the findings of this study are publicly available at https://github.com/zhizihuakai111/PPNet. Additional data are available from the corresponding authors upon reasonable request.
